# Investigations of Machining Characteristics in the Upgraded MQL-Assisted Turning of Pure Titanium Alloys Using Evolutionary Algorithms

**DOI:** 10.3390/ma12060999

**Published:** 2019-03-26

**Authors:** Gurraj Singh, Catalin Iulian Pruncu, Munish Kumar Gupta, Mozammel Mia, Aqib Mashood Khan, Muhammad Jamil, Danil Yurievich Pimenov, Binayak Sen, Vishal S. Sharma

**Affiliations:** 1 School of Mechanical Engineering, Lovely Professional University, Phagwara 144411, India; singh_gurraj@yahoo.co.in; 2 Mechanical Engineering, Imperial College London, Exhibition Rd., London SW7 2AZ, UK; 3 Mechanical Engineering, School of Engineering, University of Birmingham, Birmingham B15 2TT, UK; 4 University Center for Research & Development, Chandigarh University, Gharuan 160055, India; munishguptanit@gmail.com; 5 Mechanical and Production Engineering, Ahsanullah University of Science and Technology, Dhaka 1208, Bangladesh; mozammelmiaipe@gmail.com; 6 College of Mechanical and Electrical Engineering, Nanjing University of Aeronautics and Astronautics, Nanjing 210016, China; dr.aqib@nuaa.edu.cn (A.M.K.); engr.jamil@nuaa.edu.cn (M.J.); 7 Department of Automated Mechanical Engineering, South Ural State University, Lenin Prosp. 76, Chelyabinsk 454080, Russia; danil_u@rambler.ru; 8 Department of Production Engineering, National Institute of Technology, Agartala 799046, India; binayaksen3@gmail.com; 9 I & P Engg. Department, Dr. B.R. Ambedkar N.I.T, Jalandhar 144001, India; sharmavs@nitj.ac.in

**Keywords:** MQL, RHVT, optimization, turning, titanium, evolutionary algorithm

## Abstract

Environmental protection is the major concern of any form of manufacturing industry today. As focus has shifted towards sustainable cooling strategies, minimum quantity lubrication (MQL) has proven its usefulness. The current survey intends to make the MQL strategy more effective while improving its performance. A Ranque–Hilsch vortex tube (RHVT) was implemented into the MQL process in order to enhance the performance of the manufacturing process. The RHVT is a device that allows for separating the hot and cold air within the compressed air flows that come tangentially into the vortex chamber through the inlet nozzles. Turning tests with a unique combination of cooling technique were performed on titanium (Grade 2), where the effectiveness of the RHVT was evaluated. The surface quality measurements, forces values, and tool wear were carefully investigated. A combination of analysis of variance (ANOVA) and evolutionary techniques (particle swarm optimization (PSO), bacteria foraging optimization (BFO), and teaching learning-based optimization (TLBO)) was brought into use in order to analyze the influence of the process parameters. In the end, an appropriate correlation between PSO, BFO, and TLBO was investigated. It was shown that RHVT improved the results by nearly 15% for all of the responses, while the TLBO technique was found to be the best optimization technique, with an average time of 1.09 s and a success rate of 90%.

## 1. Introduction

In manufacturing industries, the economical and productive aspects of any production process rely directly on the manufacturing parameters [1]. As such, for machining processes, the concept of machining time, cost, and quality of product is of great concern, and this has inspired researchers to conduct comprehensive research [2]. During the cutting operation, the conversion of the mechanical energy generates heat energy [3]—this phenomenon elevates the cutting zone temperatures. This elevation varies from material to material. Factors such as the spindle speed, cutting depth, and cutting feed influence the cutting temperature [4]. Most of the machining problems and defects are directly or indirectly linked to this heating of the cutting area. In a nut shell, every machining process needs the use of a sophisticated cooling strategy. The past two decades have witnessed the use of a number of techniques, but because of economic and environmental constraints, more sustainable techniques are in demand [5]. The International Organization for Standardization’s (ISO’s) standard 14001 has been framed, with the main target of reducing environmental hazards from such industrial processes [6].

Titanium (Grade 2) is a well-known, difficult to machine super alloy utilized in numerous industries, like the aerospace and medical industries [7]. Its speciality lies in the fact that it remains stable and holds on to its original properties, even at high temperatures. The main issue related to the retention of these properties lies in the fact that it becomes a difficult-to-machine material, and as a result, it leads to issues like a poor tool life or to higher cutting forces during machining. The use of a suitable cooling strategy serves as the best possible solution for this issue. Although flooding has been used for decades, recent times have seen the development of more efficient and cost effective techniques, such as minimum quantity lubrication (MQL), which uses small amounts of a coolant or a lubricant mixed with a carrier gas [8,9,10]. In this system, the small quantities of the lubricant are directly supplied to the cutting zone. Many significant contributions have been made in the field of MQL. Attanasio et al. [11] performed a turning of 100Cr6 using MQL cooling, and studied its effects on the tool wear. It was concluded that MQL led to significant reductions in the flank wear, but did not contribute towards reducing the wear on the rake side of the tool. In a work related to the grinding of EN24 steel, Kalita et al. [12] used nano-particle enriched fluids. The specific energy and coefficient of friction were used as the result indicators. It was observed that a significant reduction of these indicators occurred as a result of increased nano-particle concentrations. It was also observed that MQL resulted in being very close to the flooding condition in terms of the residual stresses and cutting forces. The flow rate of MQL was also found to improve the results up to a particular level, after which it failed to do so [13]. On the basis of the available literature, it has been proven time and again that MQL causes better cooling of the cutting zone as a result of the better penetrating power of the mist, and also leads to a lowered wear of the tool accompanied by a better surface finish [14,15,16]. The combination of the lubrication and of the cooling effect of the fluids also reduces the friction significantly. Balan et al. [17] conducted the grinding of Inconel 751 assisted by MQL. The results saw a decent reduction in the grinding force, temperature, and the roughness. This was also accompanied by the absence of any sort of crest flattening on the material surface. In a study by Amini et al. [18], the turning of AISI 4142 was performed. The fluid flow rate and nozzle positions were varied so as to study their effect on the tool life. Optimization was carried out along with a comparison to dry machining. The MQL gave much better results. On the basis of the existing work, the area regarding the effect of a vortex tube on the MQL process is still unexplored and may lead to interesting improvements in the process.

In the current work, major upgrades have been made in the MQL technique, assisted by a Ranque–Hilsch vortex tube (RHVT). In their experimental work to validate the effectiveness of an RHVT, Selek et al. [19] made use of infra-red technology. Experiments were performed by varying the cutting depth and speed. The results obtained using the infra-red thermography showed a significant reduction in the cutting zone temperatures. Liu and Chou [20] machined hyper eutectic Al–Si alloys using RHVT cooled systems. Significant reductions in the tool wear were observed. Mia et al. [21] employed an RHVT-assisted MQL condition to improve the precision machinability of the Al 6061-T6 alloy. A few other instances are also available where the use of an RHVT has also been made, but negligible experimental studies exist where both RHVT and MQL have been combined.

In similar studies, Hussain et al. [22] worked on difficult-to-machine titanium (Ti 6246) by adding lanthanum. The new alloys that were formed gave a better machinability that was visible through the chip length. The mechanical properties remained similar to the standard alloy. Priarone et al. [23] compared MQL with other cooling techniques, while machining a gamma titanium aluminide alloy. MQL led to the best outcome. Ramana et al. [24] executed similar experimentation on Ti–6Al–4V, assisted by different types cutting tools. The MQL again outperformed its counterparts. Jawaid et al. [25] machined a Ti-6246 alloy using two types of uncoated tungsten carbide inserts—one with grain size of 0.68 µm, and the other with a grain size of 1µm. The maximum cutting speed used was 100 m/min. The outcomes were concluded using SEM analysis. In yet another exhaustive piece of work, Pervaiz et al. [26] conducted a study on the effect of the tool material on the machining properties of different materials. Uncoated carbide, coated carbide, Cubic Boron Nitride (CBN), Polycrystalline Diamond (PCD) and so on, were carefully examined for their suitability in different ranges of cutting speeds and conditions. The turning of another alloy of titanium (TC21) was performed by Wu and Guo [27]. An in-depth analysis of the cutting parameters and the tool geometry was done. The results were cross checked using a finite element method (FEM) simulation. Sharma et al. [28] also reported a lowering in the cutting temperatures using MQL. The outcomes were based on the studies of different processes using a wide range of materials. Hegab et al. [29,30] performed the machining experiments on a Ti–6Al–4V alloy under nano-fluids-assisted MQL cooling conditions. For this, the multi-walled carbon nanotubes-based nano-fluids were applied on the titanium alloy. Furthermore, Hegab et al. [31] performed a sustainability assessment on the machining of Inconel 718 under nano-fluids cooling conditions. Khatri and Jhan [32] also investigated the mechanisms of tool wear during the turning of a titanium alloy under different cooling conditions. Mia et al. [33] experimentally studied the machinability of a Ti–6Al–4V alloy under the perspective that the cutting of this alloy generates a significant amount of heat, and thus requires extreme chilling by the use of a cryogenic cooling system. Their focus was on the life cycle assessment of such machining. Maruda et al. [34,35] enhanced the performance of minimum quantity cooling lubrication by the inclusion of extreme pressure anti-wear additives in the machining of steel. They have claimed to have a better surface topography and tool wear behavior in those improved conditions. For another superalloy, Wojtewicz et al. [36] improved the performance of the minimum quantity cooling by adding MoS_2_ and graphite during the machining of Inconel. For the same group of materials, the performance of MQL was reported to improve when using hBN nanoparticles [37].

Optimization is a process that does not belong to any particular field. These techniques are used in almost all fields, including science, engineering, and commerce. Even in the machining field, the optimization process involves solving problems that are usually non-differentiable, non-linear, multimodal, multi-dimensional, stochastic, and computationally time consuming. Karkalos et al. [38] conducted a machining study for a Ti–6Al–4V ELI alloy, and developed models for the optimization of the parameters using artificial neural network and response surface methodology. The classical optimization techniques are not that reliable when solving such tasks. The natural evolutionary techniques have been found to be much more useful in solving such problems [39,40,41]. Techniques such as particle swarm optimization (PSO), bacteria foraging optimization (BFO), and teaching learning-based optimization (TLBO) are population-based, flexible, and stochastic methods.

The PSO optimization technique possesses a very simple concept and is easily implementable in a few lines of a computer code. It takes genetic algorithms and the evolution strategies into account while functioning, thus, easily solving the continuous optimization problems [42]. This technique mainly works on the concept of examining the moving trends of a group of birds. This movement is then converted into a computer-based model. The movement of these birds or of the swarm is modelled with the assistance of vectors. Each flock is referred to as an intelligent agent of particles, and then the entire space is randomly searched during several iterations. During each iteration, they look for the local best (pbest) result, and at the end of the process, they converge to the global best (gbest). Velocity updating takes place after each iteration. When the pbest gives a better result than the gbest, automatic replacement takes place. The bacteria foraging optimization technique, on the other hand, is based on the behavior during foraging by a group of bacteria [41]. Bacteria such as *E. coli* are considered when modelling the behavioral aspects. These bacteria behave in a different manner during the chemotactic processes, and move accordingly. Like the PSO process, this process treats the bacteria as intelligent agents, and their movement in search of food as the motivation to move towards the optimally best solution. The teaching learning-based algorithm is based on the effectiveness of a teacher in the learning process of the class [43]. This algorithm basically codes the teaching–learning process in a class room. In this case, the different input variables act as the different subjects given to the students, while the best solution signifies the teacher itself [44]. The application of the algorithm is generally split up into two different phases.

From the current state-of-art, it has been noted that a good amount of research is available on the machining of titanium alloys, but, on the contrary, very little research has been conducted on the Grade 2 (pure titanium). This grade has many significant properties, such as biocompatibility and resistance to corrosion. Therefore, the current work provides a proper comparative analysis of the results obtained using these evolutionary techniques (i.e., PSO, BFO, and TLBO). The results were compared with the commonly used concept of desirability. Moreover, the MQL process has not been upgraded in most of the cases. The use of RHVT alone has been made in many cases. But its combined use with MQL and the relative comparison have rarely been made for Ti machining. Thus, the importance of a lowered temperature can be observed and its effects can be quantified through this study. Machining pure titanium at a higher speed and combining the cooling technique with the effect of an RHVT may lead to significant results. A comparison of the evolutionary optimization techniques has been performed in such a domain of experiments. All of these points contribute to the uniqueness of the current study, and make advancements towards sustainable manufacturing.

## 2. Materials and Methods

### 2.1. Materials and Tools

Titanium (Grade 2), commercially pure titanium, was selected for the experiment, in the form of cylindrical bars of 150 mm in length and 50 mm in diameter. The selection of the work material was based on the lesser availability of literature related to this specific grade of titanium. Table 1 depicts the chemical composition along with more significant properties. The tool inserts that were used were uncoated carbide ones (Widia, Essen, Germany) with a nose radius of 0.8 mm, while the relief angle was 7° and the rake angle was 6°. Although sustainable manufacturing was the main consideration of the experimental work, cost effectiveness was also kept in mind, which justified the choice of the inserts used.

### 2.2. Turning Experiments and MQL Parameters

**Figure 1:**
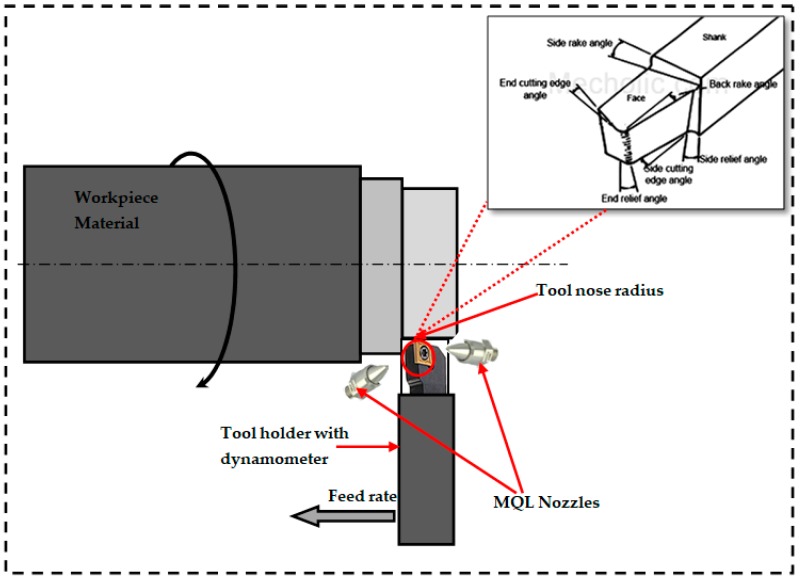
Experimental setup. MQL—minimum quantity lubrication.

CNC of make BATLIBOI and model Sprint 20TC (Gujarat, India), comprising of a motor of 11 kW with a speed range of 50–4200 rpm, was used for the experiments. The minute quantities of the coolant were dispensed through the NOGAMINI MQL system with one inlet and two outlet nozzles. The coolant used was a Rhenus FU60 ester-based oil mixed with water, in the ratio of 20:1. The cooling effect of the vortex tube was used for further cooling the pressurized air coming from the compressor. The development of the vortex tube used was Essen engineers 002H (Mumbai, India). A significant reduction in the temperature of the ambient air was obtained prior to mixing it with the coolant. The major contribution of the experimentation was to evaluate the improvements in the results obtained by using MQL with cooled air. The experimental setup is clearly shown in Figure 1. The other parameters related to MQL, such as the outlet air pressure and the flow rate, were maintained at 6 bars and 35 mL/h, respectively.

### 2.3. Measurements

The optical inspection of the worn surfaces provides the necessary information to quantitatively and qualitatively evaluate the tool surfaces [45]. This measurement is considered a non-destructive method, which can be performed with different devices, as per the accuracy requirements. The most common are the scanning electron microscope (SEM), white light interferometric (WLI) microscopy, confocal microscopy, optical diffraction technique, and the stylus methods. Therefore, the optical micrographs and surface profiles offer great information related to the dominant wear mechanisms [46].

The TeLC DKM2010 dynamometer (TeLC, Unna, Germany) was brought into use for measuring force and power. Flank wear measurements were conducted with the assistance of Leica DFC 290 tool maker’s microscope (Leica Microsystems Inc., Wetzlar, Germany). Similarly, the Mitutoyo make SJ 301 model surface tester (Kawasaki, Japan) was employed for recording the surface roughness.

One important factor that was kept in mind during the experimentation was the use of a fixed machining time. It was very clear that at different conditions, the length of cut would vary accordingly, which could alter the results. It was estimated that the wear of the insert was proportional to the machining time. The time for each experiment was fixed at 30 s for all of the machining conditions. Furthermore, to improve on the accuracy of the measured results, measurements of both the flank wear and the surface roughness were taken at three different points.

### 2.4. Experimental Design

The input parameters were all varied in three levels. Response surface methodology (RSM) was brought into use for the experimental design. The cooling condition, the sole categorical factor, was given at two levels. The rest of the parameters were selected based on the literature survey and as per the tool manufacturer’s recommendations. The Box–Behnken RSM approach suggested 26 turning experiments.

## 3. Results

The present study concentrated on four machining characteristics, namely, the average surface roughness parameter, cutting force, power consumption, and tool flank wear. Following the measurement principle, as discussed earlier, the experimental results were collected from the previous published results and shown in Table 2 [47]. Afterward, the mathematical, statistical, and optimization models were formulated. The adequacy and significance of the developed models were confirmed using an analysis of variance (ANOVA). Table 3 summarizes the results obtained after ANOVA for all of the responses.

**Figure 2:**
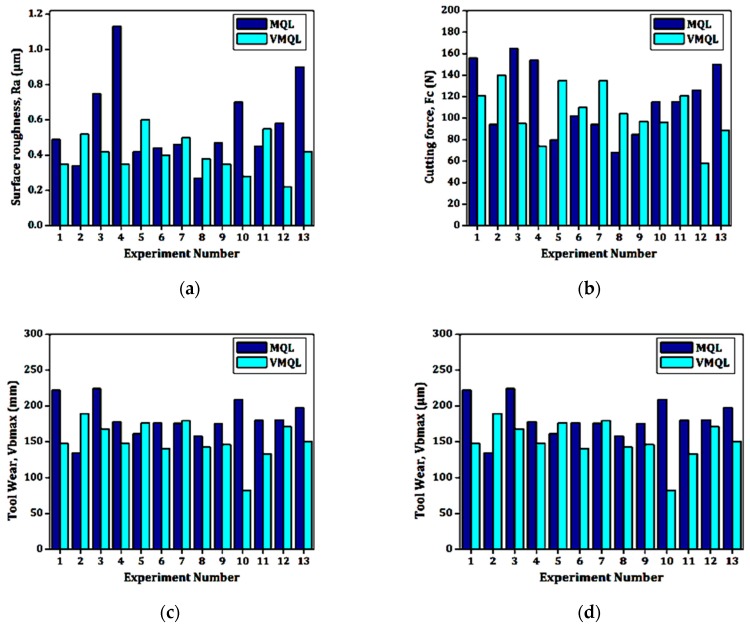
Results (**a**) MQL and vortex tube-assisted MQL(VMQL) comparisons of *R_a_*, (**b**) MQL, and VMQL comparisons of *F_c_*, (**c**) MQL and VMQL comparisons of *VBmax*, and (**d**) MQL and VMQL comparisons of cutting power (*P*).

**Figure 3:**
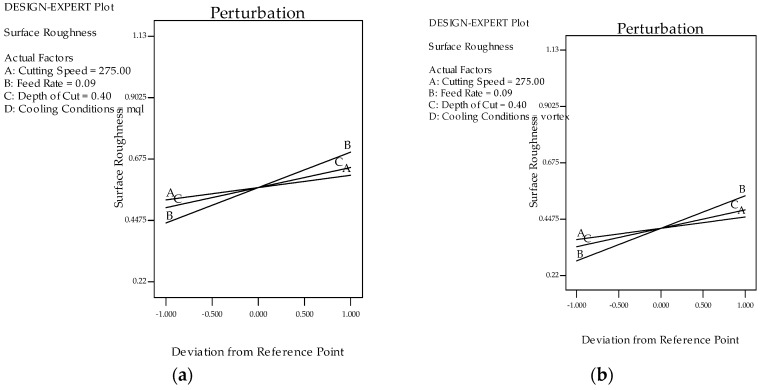

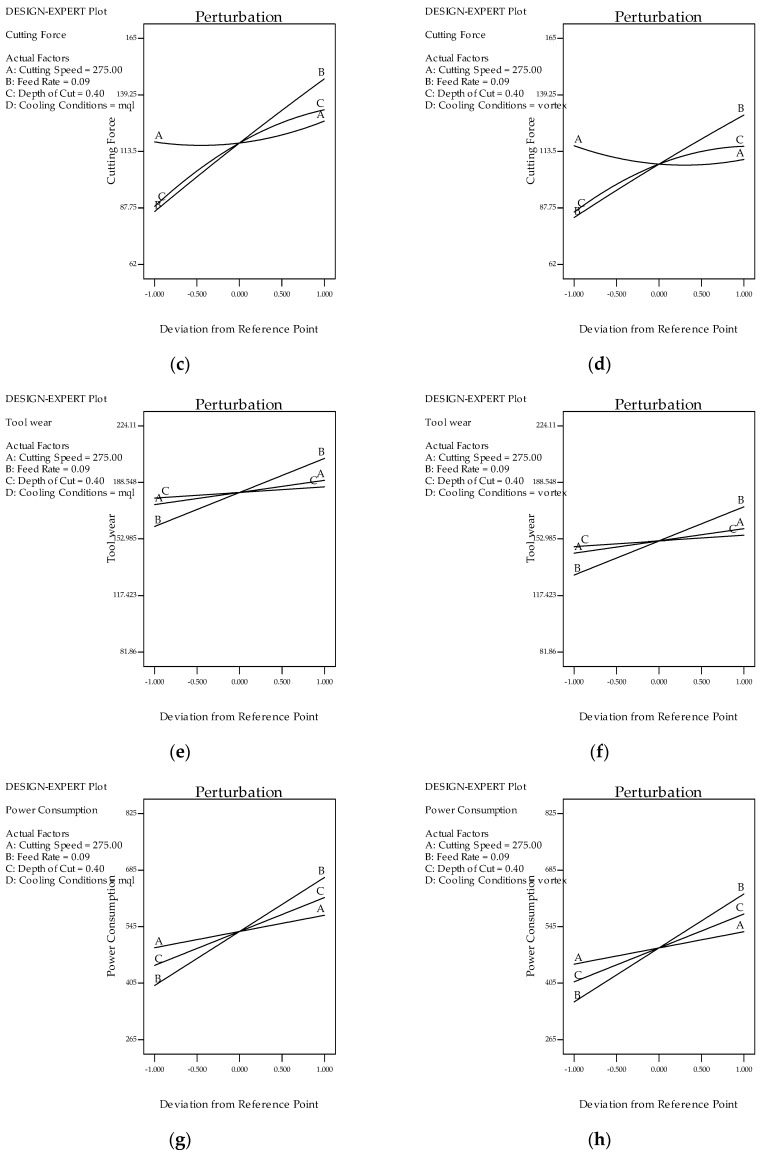
(**a**) Perturbation analysis for *R_a_* under the MQL technique; (**b**) perturbation analysis for *R_a_* under the VMQL technique; (**c**) perturbation analysis for *F_c_* under the MQL technique; (**d**) perturbation analysis for *F_c_* under the VMQL technique; (**e**) perturbation analysis for *VBmax* under MQL cooling; (**f**) perturbation analysis for *VBmax* under the VMQL technique; (**g**) perturbation analysis for *P* under thermal technique; (**h**) perturbation analysis for *P* under the VMQL technique at *v_c_* = 275 m/min, *f* = 0.09 mm/rev, and *a_e_* = 0.4 mm. (A = cutting speed; B = feed; C = depth of cut, where the X-axis represents the coded values and the Y-axis represents the selected responses) [47].

The surface finish had a dominant effect on the performance of the machined part, along with the cost considerations. This contributed towards its inclusion as one of the responses in this study. Figure 2a shows a clear-cut comparison between both of the cooling processes, when taking surface roughness into consideration. The results suggest that the vortex tube-assisted MQL (VMQL) reduced the roughness values at different combinations of input parameters. The reduction in the cutting zone temperatures due to pre-cooled air accounts for this significant reduction in the surface roughness (*R_a_*) values. The higher cooling may also have drastically reduced the thermal softening of the material, which further affected the frictional forces between the work and the insert [21]. The combined effect of all of the above-mentioned factors justifies the attained values of the surface roughness. In addition to this, Figure 2a shows the peaks formed at the higher levels of feed, which is common to both of the cooling techniques, but with different magnitudes. This led to the conclusion that VMQL becomes more effective at greater values of feed. Equations (1) and (2) were generated for the surface roughness (*R_a_*). Figure 3a,b indicates the perturbation curves for the MQL and VMQL cooling, respectively. It can be clearly seen that the feed (represented by “B” in the figure) has the maximum effect on the surface roughness (*R_a_*), while the speed (represented by “A” in the figure) and depth of cut (represented by “C” in figure) have minor effects. This observation explains and validates why the model states them as insignificant factors. It could also be seen that there was a noticeable reduction in the surface roughness when the vortex cooling was engaged.

(1) $$\mathrm{MQL}:R_{a}=-0.53+1.83\times 10^{-2}v_{c}+3.25f+0.76a_{e}$$

(2) $$\mathrm{VMQL}:\;R_{a}=-0.69+1.83\times 10^{-2}v_{c}+3.25f+0.76a_{e}$$

Figure 2b depicts the cutting force (*F_c_*) ranges recorded during experimentation with both of the cooling strategies. It can be directly stated that VMQL leads to cutting forces of lower magnitudes. The highest value of forces under the VMQL strategy was found to be nearly 16% lower than the parameter result corresponding with the MQL process. Similarly, at higher feed values, the difference recorded in the cutting forces was much more than the one recorded at lower feed levels. The depreciating cutting zone temperature clearly justified this sudden fall in the cutting forces. The lowered cutting temperature further lowered the friction at the cutting zone, which further depreciated the cutting forces. This finding directly influences the wear, surface quality, and generated heat. The machinability of any particular material can be studied through this. Figure 3c,d shows the perturbation curves for the MQL and VMQL techniques, respectively. It is clearly visible that the basic trends of all of the input parameters remain the same for both of the techniques, with VMQL giving cutting force (*F_c_*) magnitudes on the lower side. Feed (*f*) appears to be the driving parameter in effecting the cutting force (*F_c_*), while the cutting speed (*v_c_*) remains neutral. Regression Equations (3) and (4) for the cutting force (*F_c_*) for both of the cooling techniques have been shown. The results suggested by the model are incomplete agreement with the literature available. Stachurski et al. [48] concluded the role of feed (*f*) to be completely independent of the type of the cooling method applied. This result relates well to the perturbation curves obtained, showing the same trend of feed (*f*) for both of the cooling techniques. In another study, Agustina et al. [49] found the feed (*f*) to be the main factor affecting the cutting force (*F_c_*), while the speed remained ineffective. This again consolidates the results generated by the model.

Heat generation is a part of any machining process. However, the heat levels beyond specific limits may prove to be detrimental for the life of the insert and for the surface quality of the material being machined. There are three basic modes of tool failure, namely, diffusion, adhesion, or aberration. Although the widely practiced flooding technique may cause significant cooling, the penetrating effect of the MQL mist plays a leading role in reducing the flank wear (*VBmax*). Although *V_bavg_* mostly proves to be a better indicator of the wear as compared to *VBmax*, because of its statistical consistency, in the present case, as a result of higher speed levels, the wear that was recorded was highly non-uniform. Thus, *VBmax* was chosen as the wear indicator so as to eliminate misleading results. Figure 2c indicates the flank wear (*VBmax*) comparisons for both of the cooling strategies in a wide range of cutting parameters.

**Figure 4:**
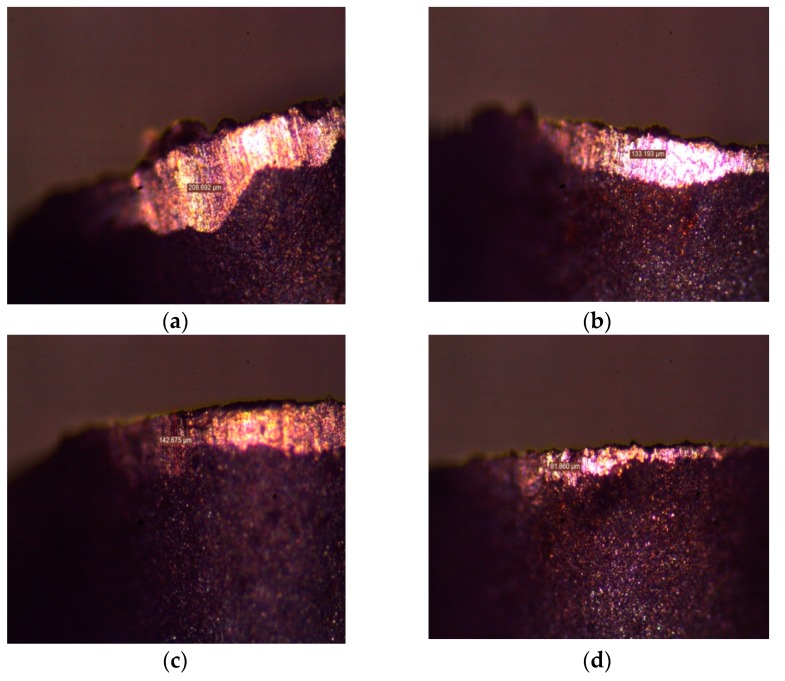
Comparison of *VBmax* for the tools found under MQL and VMQL [47]. (**a**) Condition: MQL, *v_c_* = 275 m/min, *f* = 0.13 mm/rev, *a_e_* = 0.30 mm; (**b**) Condition: MQL, *v_c_* = 250 m/min, *f* = 0.05 mm/rev and *a_e_* = 0.40 mm; (**c**) Condition: VMQL, *v_c_* = 275 m/min, *f* = 0.13 mm/rev, *a_e_* = 0.30 mm; (**d**) Condition: MQL, *v_c_* = 250 m/min, *f* = 0.05 mm/rev and *a_e_* = 0.40 mm.

Similarly, Figure 4a,b depicts the flank wear in both of the techniques at microscopic magnifications. Significant reductions in the *VBmax* values can be seen when using the VMQL technique. The perfect combination of the lubricating effect of the cutting fluid, accompanied with the chilling properties of the water component of the fluid, may have cooled the cutting zone more efficiently. It is a very well-established fact that the wear properties of the carbide tools are greatly affected by variations in temperature values [50,51]. This may have led to significant reductions in the tool wear values as a result of the reduction in the stickiness of the work material. The retention of the strength and the hardness of the tool material as a result of the lowered temperature also may have been a contributing factor. Another conclusion made was regarding the dominance of the feed at higher speeds in affecting the *VBmax* levels. The higher feed yielded higher *VBmax* values. Moreover, while evaluating the difference in the performance of the MQL and VMQL processes, the *VBmax* differences got higher at higher feed levels. As per the recorded values, VMQL may be established as the better out of the two methods. Figure 3e,f depicts the perturbation graphs of the *VBmax* for both MQL and VMQL, respectively. Feed appears to be the most vital factor, while cutting speed remains neutral. VMQL leads to a noticeable decline in the flank wear when compared with MQL. The regression Equations (3) and (4) for flank wear (*VBmax*) have been listed.

(3) $$\mathrm{MQL}\;V_{b_{\max}}=35.74+0.33v_{c}+534.26f+36.18a_{e}$$

(4) $$\mathrm{VMQL}\;V_{b_{\max}}=5.25+0.33v_{c}+534.26f+36.18a_{e}$$

While using higher speeds and rough operations, the cutting power (*P*) acts as another important parameter. Using the machine with an accurate range of power rating for specific operations also helps make the process more economical. Keeping the tool capacity under notice and selecting the right machining parameters on the basis of the power rating also helps in deciding the selection of the most apt parameters for the best material removal rate (MRR). The calculation of the cutting power is made on the basis of the Equation (5).

(5) $$P=\frac{F_{c}v_{c}}{60}$$

We also know that $F_{c}=k_{c}a_{e}f$, where *k_c_* is the coefficient of specific energy (N/mm^2^). Thus, the cutting power can be calculated as shown in Equation (6).

(6) $$P=\frac{k_{c}a_{e}fv_{c}}{60}$$

The generated equation proves that the cutting power (*P*) directly depends on all of the process parameters. Figure 2d indicates the cutting power (*P*) comparisons between the MQL and VMQL techniques. It can be clearly visualized that the pattern followed by the power consumption is similar to the one formed by the cutting forces. As explained earlier, the temperature reduction caused the friction to fall, thus reducing the power consumed. Reduced flank wear also plays a role in reducing the power consumed. Figure 3g,h shows the perturbation curves for both of the techniques. The power consumption follows the same path as the force (*F_c_*), as the feed (*f*) is a dominant parameter and the speed (*v_c_*) remains insignificant. The model shows a similar decrease in the power consumption values, thus confirming the experimental results. The regression Equations (6) and (7) were obtained for the power consumption (*P*). Hence, the use of the vortex principle for cooling led to reduced power consumption.

(7) $$\mathrm{MQL}\;P=-544.12+1.63v_{c}+3365.35f+843.03a_{e}$$

(8) $$\mathrm{VMQL}\;P=-583.40+1.63v_{c}+3365.35f+843.03a_{e}$$

### 3.1. Optimization

The result analysis process was followed by the optimization process. Optimization was carried out in a comparative manner by comparing the PSO, BFO, TLBO, and desirability methods. The combined objective (C.O.) equation was calculated as per Equation (8).

(9) $$Minimum\;C.O.=W_{t_{1}}\frac{R_{a}}{R_{a_{\min}}}+W_{t_{2}}\frac{F_{c}}{F_{c_{\min}}}+W_{t_{3}}\frac{V_{b\max}}{V_{b\max_{\min}}}+W_{t4}\frac{P}{P_{\min}}$$

The weights were assigned in such a way that their summation added on to unity. The finalization of these weights may also be done using random combinations. The variation in the weightings can be allowed to vary from 0.1 to 0.5, in such a way that their summation remains unified. One similar result was presented in the current work, where each of the response parameters was assigned an equal weight of 0.25. The result yielded by the combined objective (C.O.) approach may lead to outcomes totally different from the individual ones, which points to the pros of employing this technique.

### 3.2. Parametric Optimization Using Desirability Function Approach

Apart from the evolutionary algorithms, the desirability technique was also used for optimizing the results. It is usually performed in both single and multiple objective functions. Unlike other single response optimization strategies, this technique prevents the response clashing. The variation of the input parameters was done in order to allow them to take any value within the given range. The output, that is, the combined objective in this case, was chosen to be minimized in order to achieve optimum results. Table 4 lists the top three desirable results. It could be concluded that VMQL leads to the most optimum results, where the values of feed, cutting depth, and cutting speed were 0.07 mm/rev, 0.31 mm, and 255 m/min respectively.

### 3.3. Parametric Optimization Using PSO, BFO, and TLBO

To conduct optimization using these techniques, the regression analysis was used to obtain the combined objective function equations. This was followed by the use of MATLAB for implementing the obtained fitness function. The same procedure was applied for all three of the techniques, while the combined objective was minimized each time. Equation (9) shows the generated objective function.

(10) $$C.O.=+2.01+0.12v_{c}+0.46f+0.26a_{e}-0.16CC$$

**Figure 5:**
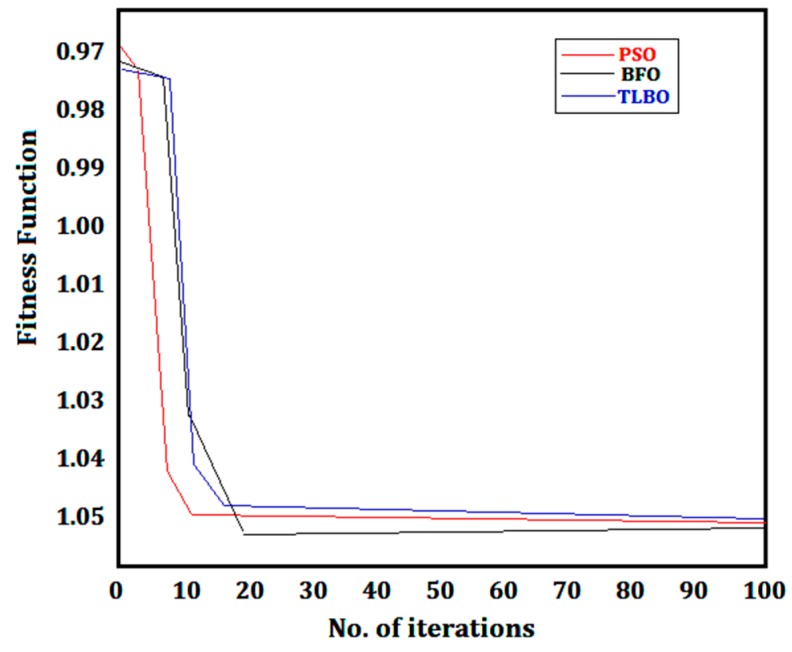
Convergence graph comparisons.

Table 5 depicts the developed PSO model with the constraints. Figure 5 throws light on the convergence characteristics obtained by these techniques for the current experimentation. The success of the technique lies in the iterations performed. The higher the number of iterations, the better the learning rate of the intelligent agents is, and the search space is explored more efficiently. The main aim of the techniques is to converge towards the global best values within a minimum time, thus saving valuable time. Moreover, the maximum magnitude of the particle velocity is also controlled using an equation. The control is important, because an uncontrolled velocity may lead to velocities in the opposite (negative) direction.

Similarly, in case of the BFO, the parameters were selected on the basis of the available literature. *S* represents the number of bacteria and was kept at 55. The number of chemotactic steps was taken as 120, while the reproduction steps were maintained at 5. Like the iterations in the PSO process, the numbers of chemotactic steps controlled the convergence characteristics of the process. The optimal results were obtained by fixing the probability of elimination and dispersal at 0.1 as shown in Table 6. Figure 5 also depicts the convergence graph for BFO, and it accounts for its stability and precision. The TLBO technique consists of a continuous repetition of the parameters (like the number of iterations or the size of the population). Such a continuous process ultimately leads to an optimal solution. The updating of the solutions takes place in both the learner and the teacher phases. Moreover, while commencing with the step for the duplication elimination, the existence of duplicate solutions leads to their random modification. Therefore, the main calculations in TLBO are as follows: (2 × Iteration number × size of population) + (duplicative elimination). The counting of the function evaluations is accomplished through the above-mentioned formula. For the present work, 2000 evaluations were performed in order to eliminate the duplicity.

Table 7 depicts the different values of the C.O. function. There were 100 runs that were conducted to calculate the success rate and the consumed time. Each method disclosed similar results. The most suitable of the techniques was TLBO, with a success rate of 90% and an average time of 1.10 s, whereas PSO and BFO consumed 6.5 and 14.63 s with a success rate of 62% and 53%, respectively. The success rate of the TLBO was high and the average time taken was much less compared with PSO and BFO. This is because TLBO is free from any algorithm parameters and converges within 20 iterations, whereas PSO and BFO take 100 iterations. For example, in PSO, the total number of particles was taken as 46, and for 100 iterations, the total number of fitness evaluations will be 46 × 100 = 4600. In BFO, a total of 60 bacteria were considered, with 100 chemotactic, 4 reproduction, and 5 elimination and dispersal events. Thus, the total fitness evaluations were computed as 60 × 100 = 6000 before the first reproduction stage. There were a total of four reproduction stages, thus, the total evaluations became 4 × 6000 = 24,000 before the elimination and dispersal event. Then according to 0.1 *P_ed_* (probability of elimination and dispersal), 10% of the bacteria among the total 60 bacteria were further dispersed randomly, so as to avoid the chance of being trapped in the local minima. These bacteria further enhanced the number of fitness evaluations, thus the total fitness evaluations were more than 24,000. In the case of TLBO, only 1000 steps were involved. This explains the higher evaluation in PSO and BFO when compared to TLBO. Moreover, from the convergence characteristics graphs, it is clearly seen that the convergence of TLBO mimics good convergence characteristics, as compared with PSO and BFO (shown in Figure 5). Validation experiments were also performed on the basis of the obtained results. Table 8 also shows that the optimum values obtained using PSO, BFO, and TLBO are slightly higher when compared with the ones obtained with desirability. This leads to the conclusion regarding the effectiveness of the evolutionary algorithms when compared to the desirability method when finding the optimized parameters. In addition to the optimization process, confirmation experiments were also conducted in order to confirm the generated results, as listed in Table 8. From the given results, it has been noted that a cutting speed of 255 m/min, feed rate of 0.07 mm/rev, depth of cut of 0.31, and VMQL conditions are the ideal conditions during the machining of titanium alloy.

## 4. Conclusions

The present study concentrated on the machinability investigation and on the optimization of the turning parameter of the difficult-to-cut pure Ti alloy under traditional MQL and upgraded vortex tube-assisted MQL, with respect to the surface roughness, power consumption, tool wear, and cutting forces. This study highlights that the use of the VMQL technique allows for obtaining a highly improved surface finish (*R_a_*) with a reduction that varies from 15%–18%, when compared with the MQL process alone. Furthermore, reductions in the cutting force (*F_c_*) and the power consumed (*P*) were achieved. The feed rate was identified as the most dominant of the input parameters. Flank wear (*VBmax*) witnessed a downward trend when measured for the VMQL process. Hence, the manufacturing industries of Ti can extract benefits from this survey by using the new addition of RHVT, resulting in economic and sustainable benefits. The experimental results were compared to the mathematical models generated by ANOVA. A robust agreement was achieved between the experimental results and the mathematical models. The evolutionary optimization strategies, namely, the PSO, BFO, and TLBO techniques, were compared to the desirability technique, while the VMQL technique seems to be a better one. The TLBO technique was concluded as the best one amongst the rest, giving a high success rate of 90% and using the minimum time. These methods are proven to be effective, especially from the perspective of the smart and intelligent controlling of manufacturing systems as part of the Industry 4.0. Apart from the mentioned advantages, the cooling technique led to making the process greener, through the simple addition of an RHVT.

## Figures and Tables

**Table 1:** Composition and properties (provided by: Maharaja Associates, Mumbai, India).

Composition or Properties	Data
C	<0.12%
Fe	<0.33%
H	<0.015%
N	<0.034%
O	<0.24%
Ti (balance) (%)	98.8%
Density	4500 Kg/m^3^
Specific heat	520 J/(Kg·K)
Thermal conductivity	16 W/(m·K)

**Table 2:** Experimental results [47]. MQL—minimum quantity lubrication; VMQL—vortex tube-assisted MQL.

Serial Number	Cutting Speed, *v_c_* (m/min)	Feed Rate, *f* (mm/rev)	Depth of Cut, *a_e_* (mm)	Cooling Condition	Surface Roughness, *R_a_* (µm)	Cutting Force, *F_c_* (N)	Power Consumption (Watts)	Tool wear, *VBmax* (µm)
1	0	0	0	MQL	0.49	156	716	221.82
2	0	1	1	MQL	0.34	94	390	134.39
3	−1	−1	0	MQL	0.75	165	825	224.11
4	1	1	0	MQL	1.13	154	704	177.86
5	0	0	0	MQL	0.42	80	399	160.95
6	1	0	1	MQL	0.44	102	467	176
7	1	−1	0	MQL	0.46	94	468	175.82
8	0	−1	1	MQL	0.27	68	344	157.57
9	1	0	−1	MQL	0.47	85	354	175.17
10	0	0	0	MQL	0.7	115	528	208.71
11	0	−1	−1	MQL	0.45	115	526	179.64
12	0	0	0	MQL	0.58	126	525	180.25
13	−1	0	−1	MQL	0.9	150	683	197.4
14	0	1	−1	VMQL	0.35	121	504	147.54
15	0	0	0	VMQL	0.52	140	699	189.14
16	−1	0	1	VMQL	0.42	95	435	168.06
17	−1	1	0	VMQL	0.35	74	372	148
18	−1	0	1	VMQL	0.6	135	610	176.17
19	1	1	0	VMQL	0.4	110	506	140.24
20	0	0	0	VMQL	0.5	135	675	179.24
21	0	−1	1	VMQL	0.38	104	479	142.65
22	1	−1	0	VMQL	0.35	97	404	145.74
23	−1	1	0	VMQL	0.28	96	402	81.86
24	0	0	0	VMQL	0.55	121	604	133.32
25	0	1	1	VMQL	0.22	58	265	170.84
26	0	1	−1	VMQL	0.42	89	444	150.41

**Table 3:** Analysis of variance (ANOVA) table.

Factors	Responses			
	Cutting Force, *F_c_* (N)	Tool Wear, *VBmax* (µm)	Surface Roughness, *R_a_* (µm)	Power Consumption (Watts)
R-square	0.8867	0.6362	0.56	0.8957
Adjusted R-Square	0.8651	0.5669	0.4762	0.8758
Predicted R-Square	0.8225	0.4312	0.3148	0.8364
Adequate Decision	22.129	10.164	8.97	22.089
Model F-Value	41.08	9.18	6.68	45.07

**Table 4:** Optimized results (desirability approach).

Cutting Speed (m/min)	Feed Rate (mm/rev)	Depth of Cut (mm)	Cooling Condition	Combined Objective	Desirability
255	0.07	0.31	VMQL	1.26708	0.906
250	0.05	0.40	VMQL	1.27166	0.906

**Table 5:** Particle swarm optimization (PSO) parameters.

Parameters	Values
Number of variates	5
Number of particles	55
Number of iterations	120
Inertial weight (W)	0.7
Rate of learning	-
C_1max_ = C_2max_	1.7
C_1min_ = C_2min_	0.5
C_1_ = C_2_ = C_min_ + R × (C_max_ − C_min_)	Where R = current iterations/total iterations
X_min_	[250 0.05 0.3 MQL]
X_max_	[300 0.13 0.5 VRHVT]

**Table 6:** Parameters of bacteria foraging optimization (BFO).

Input Parameters	Value of Parameters
p, search area dimension	4
S, number of bacteria	55
N_c_, number of chemotactic steps	120
N_re_, number of reproduction steps	5
N_ed_, number of elimination-dispersal events	5
N_s_, maximum swim steps	4
P_ed_, probability of elimination and dispersal	0.1
C_max,_ run length (maximum)	0.2
C_min_, run length (minimum)	0.01
$R_{U}^{P}$, Upper search space constraints	[250 0.05 0.3 MQL]
$R_{L}^{P}$, Lower search space constraints	[300 0.13 0.5 RHVT]
d_attract_ = d_repellent_, depth of attractant and repellent signals	0.1
w_attract_, attractant signal width	0.1
w_repellent_, repellent signal width	0.1

**Table 7:** Comparison of combined objective values by PSO, BFO, teaching learning-based optimization (TLBO), and desirability function approach.

Technique	Best Case	Worst Case	Average Reading	Time Taken	Success (%)
PSO	1.049	1.056	1.053	6.40	60
BFO	1.056	1.057	1.058	14.70	50
TLBO	1.042	1.049	1.045	1.09	90
Desirability Function		1.267			

**Table 8:** Optimal parameter settings.

Parameters	Cutting Speed (m/min)	Feed Rate (mm/rev)	Depth of Cut (mm)	Cooling Mode	CO
PSO	255	0.07	0.31	VMQL	1.053
BFO	255	0.07	0.31	VMQL	1.058
TLBO	255	0.07	0.31	VMQL	1.044
Desirability	255	0.07	0.31	VMQL	1.267
Experimental	255	0.07	0.31	VMQL	1.042
